# The mesocortical dopaminergic system cannot explain hyperactivity in an animal model of attention deficit hyperactivity disorder (ADHD)- Spontaneously hypertensive rats (SHR)

**DOI:** 10.1186/s42826-023-00172-5

**Published:** 2023-09-14

**Authors:** Aysegul Gungor Aydin, Esat Adiguzel

**Affiliations:** 1 Department of Psychology, Rutgers University-New Brunswick, Piscataway, NJ 08854 USA; 2 Department of Anatomy, Faculty of Medicine, Pamukkale University, 20070 Denizli, Turkey; 3 Department of Neuroscience, Institute of Health Sciences, Pamukkale University, 20070 Denizli, Turkey

**Keywords:** Attention deficit hyperactivity disorder, Dopamine, Ventral tegmental area, Prefrontal cortex, Spontaneously hypertensive rat, Tyrosine hydroxylase

## Abstract

**Background:**

Attention deficit hyperactivity disorder (ADHD) is one of the most prevalent neuropsychiatric disorders with morphological brain abnormalities. There is a growing body of evidence that abnormalities in the dopaminergic system may account for ADHD pathogenesis. However, it is not clear whether the dopaminergic system is hyper or hypoactive. To determine whether the DA neurons and/or axons deficiency might be the cause of the postulated dopaminergic hypofunction in spontaneously hypertensive rats (SHR, animal model of ADHD), this study examined the dopaminergic neurons and fibers in the brain tissues of SHRs and Wistar Kyoto rats (WKY, control animals). Here, we performed immunohistochemical tyrosine hydroxylase (TH) and dopamine-beta-hydroxylase (DBH) staining on brain sections collected on juveniles from SHR and WKY. Moreover, behavioral testing to examine the hyperactivity in the open field area was also elucidated.

**Results:**

The mesocortical dopaminergic system appears to be normal in juvenile SHR, as suggested by (i) no alteration in the area density of TH-immunoreactive (TH-ir) dopaminergic neurons in the ventral tegmental area (VTA), (ii) no alterations in the volume density of TH-ir fibers in layer I of the prelimbic (PrL) subregion of medial PFC (mPFC), (iii) no alteration in the percentage of TH-ir dopaminergic fibers in layer I of the PrL subregion of mPFC as revealed by TH and/or DBH immunoreactivity. Furthermore, the SHR showed increased locomotor activity than WKY in the open field test.

**Conclusions:**

The demonstration of no alteration in mesocortical dopaminergic neurons and fiber in SHR raises some concern about the position of SHR as an animal model of the inattentive subtype of ADHD. However, these results strengthen this strain as an animal model of hyperactive/impulsive subtype ADHD for future studies that may elucidate the underlying mechanism mediating hyperactivity and test various treatment strategies.

## Background

Attention -deficit hyperactivity disorder (ADHD) is one of the most prevalent neuropsychiatric disorders of childhood, characterized by locomotor hyperactivity, impaired sustained attention, impulsivity, and distractibility [1]. The worldwide prevalence of ADHD is 5.29%, also the prevalence of childhood and adulthood ADHD is 5–10% and 4%, respectively [2–4]. Multiple hypotheses have been proposed for the etiology of ADHD [5–9] but one that has stood the test of time is dopamine (DA) deficit theory [10]. Previous studies have attributed the pathophysiological mechanism of ADHD to disturbances in the dopaminergic system [11, 12].

There is a significant amount of data in the literature suggesting a DA-ADHD association. It is reported that the alterations in DA signaling in both individuals (Jucaite et al. 2005; Ludolph et al. 2008; Volkow et al. 2009) and rodents [13–15] can be correlated to ADHD symptoms. However, the specific neurobiological mechanisms underlying this disorder still remain unclear. ADHD may result from deficits in the dopaminergic system, especially the mesocortical one, which projects from the ventral tegmental area (VTA) to the PFC [16, 17]. The mesocortical DA inputs exert an inhibitory effect on the activity of PFC neurons [18, 19]. Due to its strong inhibitory effect on prefrontal cortical neurons, dysfunction of the mesocortical dopaminergic projections is involved in the behavioral problems of children with ADHD [19]. Animal studies showed that lesions of the mesocortical projection to PFC lead to behavioral problems similar to those seen in ADHD [20]. However, studies on animal models aim at verifying the current postulate of a dysfunctional dopaminergic system at the level of neuroanatomy in ADHD are scarce. Thus, investigating the neuroanatomical changes in the mesocortical dopaminergic system provides a basis for future studies to explore the underlying mechanisms of hyperactivity and test different treatment strategies.

The medial prefrontal cortex (mPFC) of the rat is most likely comparable to that of humans [21–23]. Neurochemical, pharmacological, genetic, and imaging studies in human and animal models highlight the distruptive effect of catecholamine dysfunction and particularly DA in cortical brain structures such as the PFC in the neurobiology of ADHD [24–32]. The PFC requires optimal levels of catecholamine neurotransmitters norepinephrine (NE) and DA for regulating PFC-dependent executive functions that are often reported to be suboptimal in ADHD patients [33–35]. Hence, attentional, psychomotor, reinforcing, and rewarding behaviors in which DA plays an essential modulatory role are deficient in ADHD [36, 37]. PFC receives dopaminergic innervation from the VTA and substantia nigra [38]. These dopaminergic innervations in frontal cortices target layer I and are a conserved feature across rodents [39, 40]. Layer I is the primary target of projections relaying top-down signals [35] and is the most densely innervated layer by dopaminergic fibers across all labeled cortices [41]. Thus examining layer I innervation of DA neurons from VTA to PFC might contribute to understanding the mechanisms underlying ADHD.

The Spontaneously hypertensive rat (SHR) is currently the best-validated animal model of ADHD based on behavioral, genetic, and neurobiological data [42–44]. SHRs are normotensive at birth and gradually develop increases in blood pressure beginning at 6–7 weeks of age and reach a stable level of hypertension by 17–19 weeks of age [45–47]. SHR shows overactivity under the control of a fixed-interval operant reinforcement schedule [42, 48], increased behavioral variability [49] and problems with cognitive impulsiveness [50] similar to that of ADHD children. Abnormalities in DAT-1 gene expression are seen both in ADHD individuals [51, 52] and in the SHR [53]. Furthermore, chronic oral methylphenidate at therapeutically relevant doses improves behavioral and cognitive deficits in SHR [54, 55]. In addition, SHR exhibits hyperactivity, as seen in ADHD children, in the open field test [56].

Several studies have suggested that either too little, or too much D1 receptor stimulation impairs PFC function [57–60]. Clinical and experimental evidence suggests the involvement of DA systems, especially the mesocortical systems, in ADHD [16]. The mesocortical DA pathway, originating from the VTA and projecting to the PFC, is involved in cognitive functioning [61, 62]. However, it is still unclear whether the mesocortical dopaminergic system is hyper or hypo-functioning. Therefore, in the present study, we tested the hypothesis that SHR shows hypofunctional dopaminergic system by investigating the neuroanatomical changes in the mesocortical dopaminergic system. We addressed this hypothesis by setting out to determine whether the DA neurons and/or axons deficiency might be the cause of the postulated dopaminergic hypofunction in SHR. Thus, immunohistochemical analysis was carried out on neurons and fibers of mesocortical DA systems of the SHR and WKY. WKY rats are considered a proper control for the SHR as they were both established from some paternal, normotensive Wistar stock [43]. Also, our study aimed to analyze the dopaminergic neurons and fibers in SHR and WKY to search for neuroanatomical evidence that might explain the hypofunctional dopaminergic system and assess the face validity of this animal model in the open field test.

## Results

### Systolic blood pressure

**Fig. 1:**
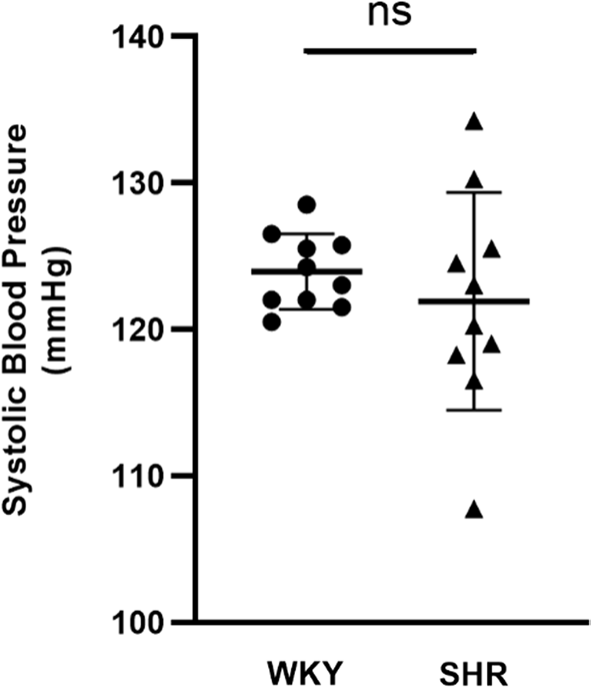
Tail cuff systolic blood pressure measurements of the WKY and SHR (n = 10) animals at the age of 4 weeks. Values are expressed as mean ± SD (*p* > 0.05, two-tailed unpaired t-test). No difference in SBP of SHR groups from corresponding WKY groups. SBP, systolic blood pressure

To test whether or not SHR is hypertensive SBP measurements are presented in Table 1. Non-invasive SBP measurement confirmed normotension at 4 weeks of age in the SHR group (121.9 ± 7.43 mmHg) and in the age- and sex-matched WKY group (124 ± 2.57 mmHg). The results revealed that there was no significant difference in SBP between the SHR (n = 10) and WKY (n = 10) groups (*p* > 0.05) (Fig. 1).

**Table 1:** Systolic blood pressure from WKY and SHR rats

Groups	1st measurement (mmHg)	2nd measurement (mmHg)	3rd measurement (mmHg)	4th measurement (mmHg)
WKY (n = 10)	123.6 ± 5.30	125.5 ± 5.61	124.6 ± 4.50	122.1 ± 2.02
SHR (n = 10)	121.9 ± 10.26	119.5 ± 9.20	125.8 ± 8.15	120.5 ± 10.71

The table shows four different SBP measurements from the groups

Mean data ± SD are presented for SHR and WKY animals at the age of 4 weeks

SHR, spontaneously hypertensive rats; WKY, Wistar Kyoto rats (*p* > 0.05)

### Open field test

**Fig. 2:**
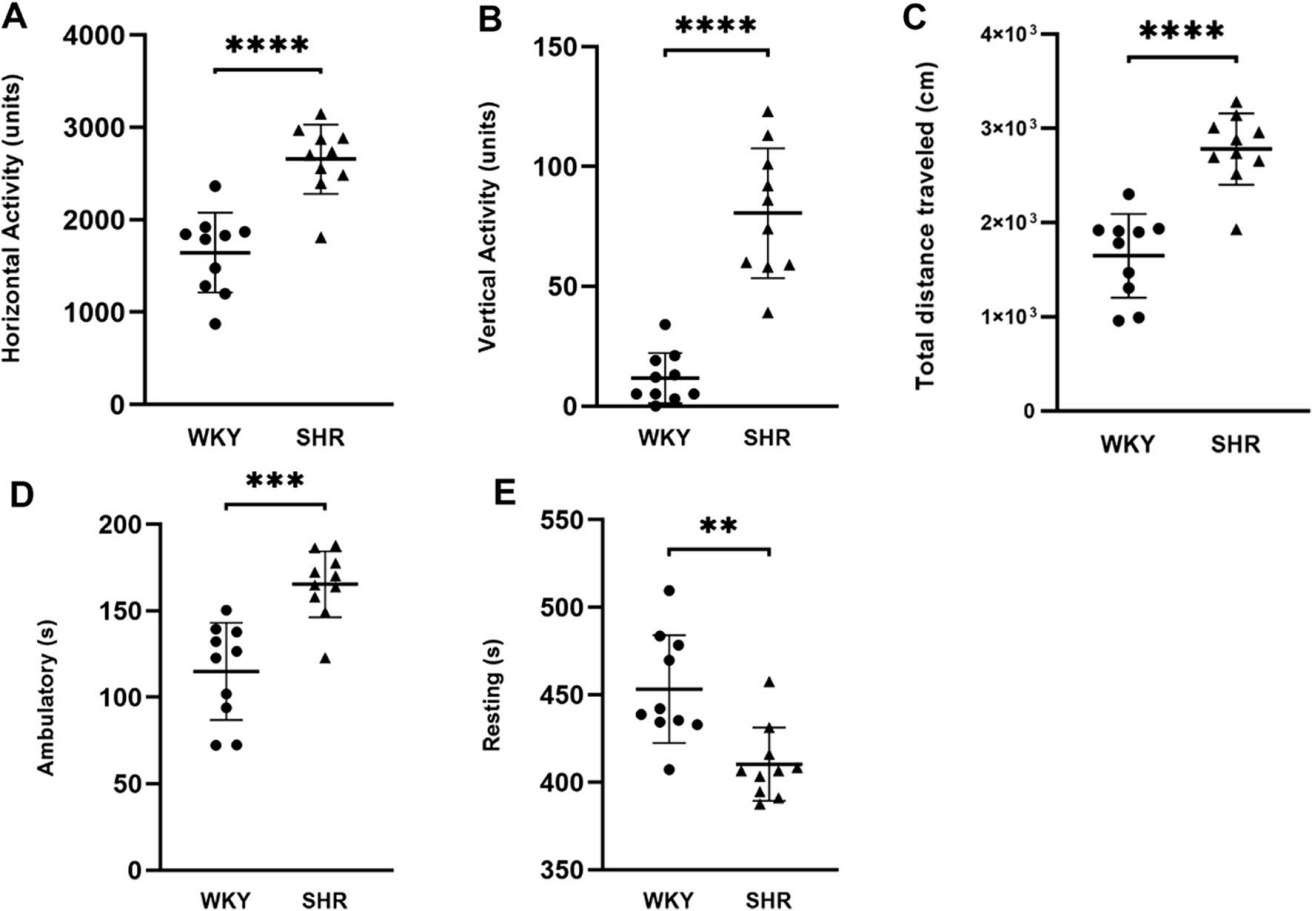
Increased locomotor activity in SHR rats compared to WKY rats in the open field test (n = 10 in each group). **A** Horizontal activity (arbitrary units), **B** vertical activity (arbitrary units), **C** total distance traveled (cm), **D** ambulatory (s), **E** Resting (s). All data are presented as mean ± SD. SHR and WKY rats were compared with an independent sample T-test. A *p* value < 0.05 was considered significant. **p < 0.01, ***p < 0.001, ****p < 0.0001

To assess the locomotor activity in SHR, an open field test was performed. Ten rats per strain were tested in this experiment. When placed into the open-field arena for the first time, i.e. non-habituated, SHR rats were significantly more active compared to WKY rats. The results are shown in Fig. 2, SHR rats showed significantly more horizontal (Fig. 2A, *p* < 0.0001, t = 5.574) and vertical activity (Fig. 2B *p* < 0.0001, t = 7.495), increased distance traveled (Fig. 2C *p* < 0.0001, t = 6.118), higher ambulatory activity (Fig. 2D *p* < 0.001, t = 4.689), and less immobility time (Fig. 2E *p* < 0.01, t = 3.650) than WKY rats. This finding indicates that SHR rats exhibited hyperactivity as revealed by the Open field test.

### Area density of TH-ir dopaminergic neurons in VTA

**Fig. 3:**
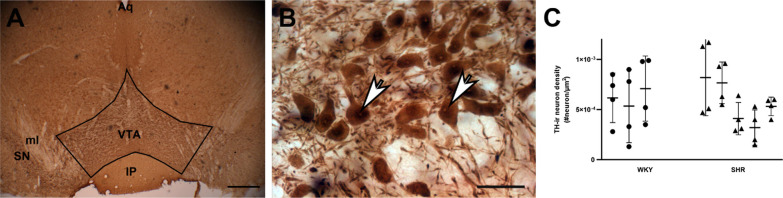
TH-ir neuron density in the VTA. **A** A coronal brain section through the VTA, immunostained for TH. Black lines mark putative anatomical boundaries of the VTA. **B** Higher magnification view of the region outlined with a square in panel A, revealing TH-immunoreactivity filling the cytoplasm of the VTA cells (white arrows) and the segments of dendrites emanating from labeled cells. Scale bar = 400 µm in A, and 20 µm in B. **C** Volumetric density of TH-ir cells in WKY (black bars) and SHR (white bars) animals (n = 3 WKY and 5 SHR; 4 sections per animal; *p* > 0.05 in Nested t-test). IP: interpeduncular nucleus; ml: medial lemniscus; SN: substantia nigra; Aq: aquaeductus cerebri

To assess whether there are fewer TH-ir neurons in VTA in the SHR, we measured the area density of TH-ir dopaminergic neurons Fig. 3). The mean area density of neurons in the VTA was 6.2 ± 2.9(× 10^−3^) cell per mm^2^ (mean ± SD) for the control group, 5.7 ± 2.8 (× 10^−3^) cell per mm^2^ for the SHR group. The difference between the control and SHR groups was not statistically significant (*p* > 0.05 from the Nested t-test).

### Volume density of TH-ir fibers in layer I of the PrL subregion of mPFC

**Fig. 4:**
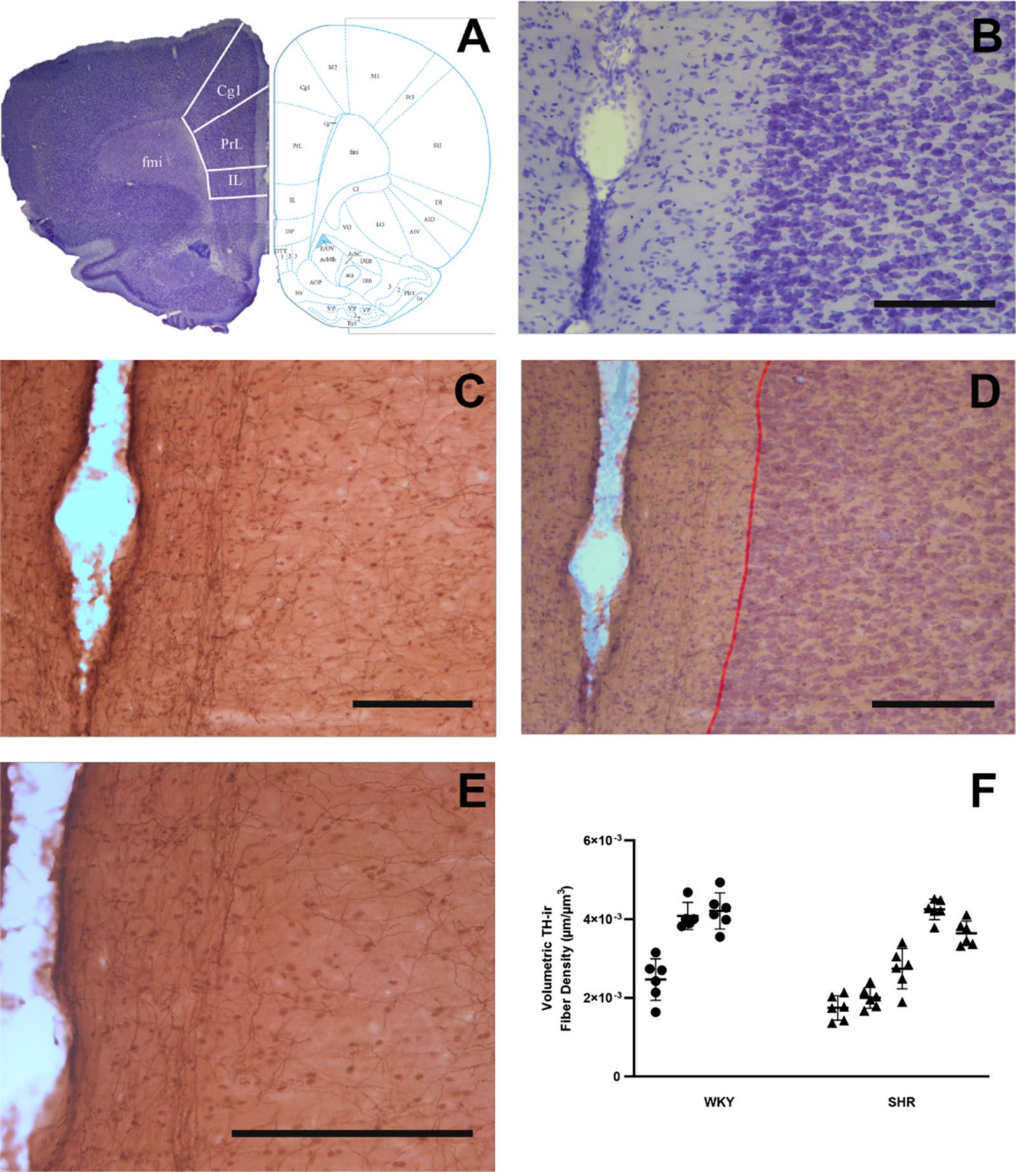
TH-ir fiber density in layer I of the PrL subregion of mPFC. **A**, **B** Coronal brain sections are used to identify the PrL of the mPFC (at around Bregma 3.00) on adjacent sections that are immunostained for TH (**C**, **E**). TH-ir fibers are encountered at all layers of the cortex, while those in Layer I are selectively oriented parallel to the pila surface (**D**, **E**). TH-ir fiber density measurements were confined in layer I, between the pial surface and Layers I and II border (red line). All scale bars = 250 µm. **F** The volumetric density of fibers in Layer I of the mPFC-PrL of SHR and WKY rats (n = 3 WKY and 5 SHR; 6 sections per animal; *p* > 0.05 in Nested t-test). PrL: prelimbic area; IL: infralimbic area; Cg1: anterior cingulate cortex

To determine whether the SHR has decreased TH-ir fibers in mPFC, we calculated the volume density of TH-ir fibers. The mean volume density of TH-ir fibers in layer I of the PrL subregion of mPFC was 0.0036 ± 0.0009 µm/µm^3^ (mean ± SD) for the control group, 0.0029 ± 0.0010 µm/µm^3^ for the SHR group (Fig. 4). The mean volume density of TH-ir fibers was significantly lower in SHR (*p* > 0.05 from Nested t-test).

### Percentage of TH-ir dopaminergic fibers in the mPFC

**Fig. 5:**
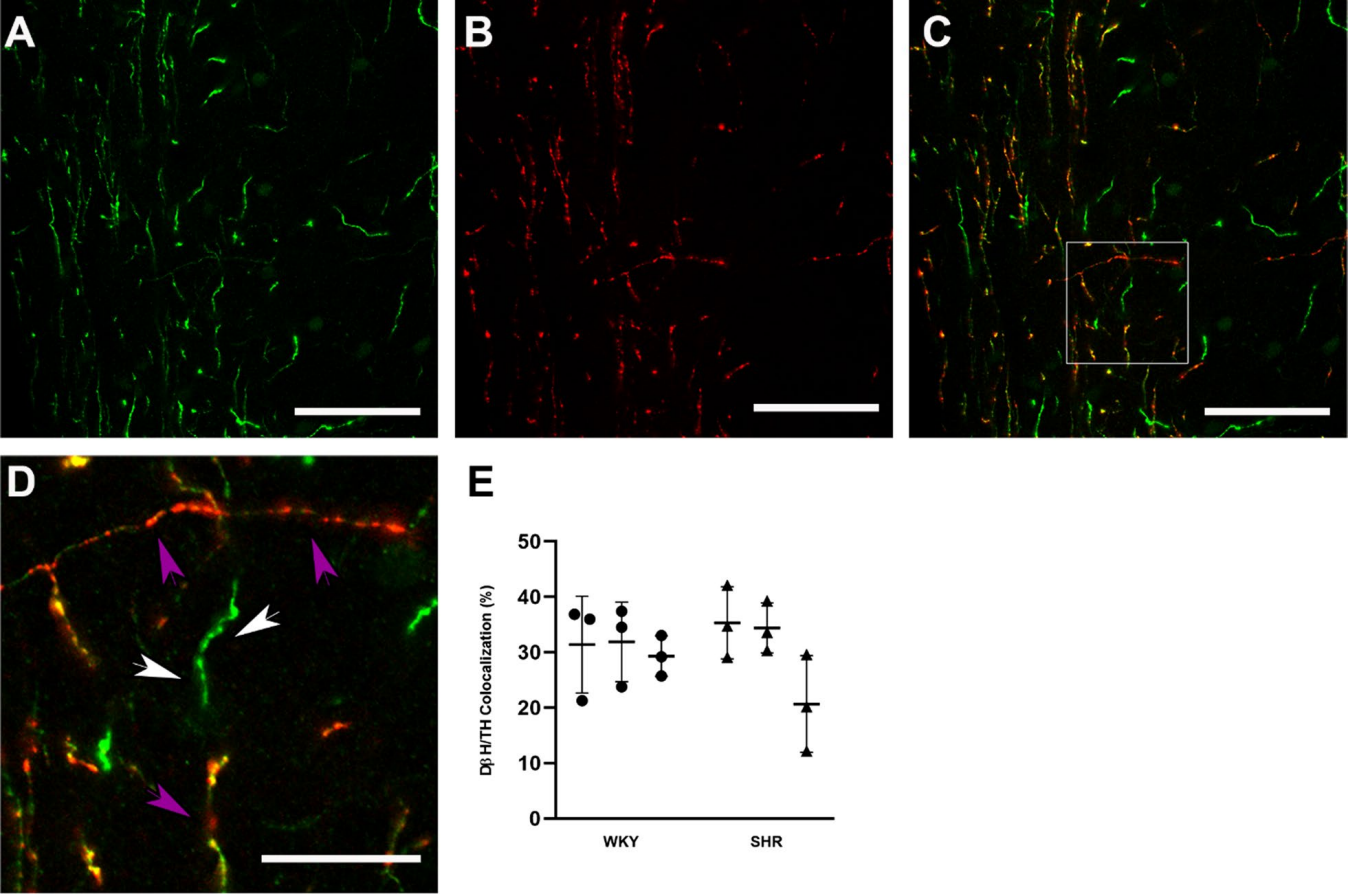
Colocalization analysis in layer I of the PrL subregion of mPFC. The axons immunolabeled for TH (green in **A**) and DBH (red in **B**) are imaged in the mPFC of a control brain. Merging of two images (**C**, **D**) reveals the presence of DBH (red; purple arrows) in TH (green; white arrows) axons and these dual labeled axons display patches of yellow fluorescence. Panel **D** is the high magnification of the area marked with a square frame in panel **C**. Note that all DBH-ir axons (purple arrows) are also TH-ir, but not all TH-ir (white arrows) are also DBH-ir. Scale bars: 50 µm in **A**–**C** and 15 µm in **D**. **E** The percentage of TH-ir axons in the mPFC. The measurements were obtained from 3 animals in each group 3 sections per animal; *p* > 0.05 in the Nested t-test). No significant difference was found between the groups

Because the TH-ir represents not only dopaminergic but also noradrenergic fibers from locus cereolous, we used dual; staining with TH and DBH antibodies to reveal the proportion of TH fibers that are not noradrenergic. Double immunofluorescent staining and colocalization analysis was performed on three slices from 3 areas of 3 animals. The percent ratio of TH-ir fibers that are not DBH + (thus, dopaminergic) in layer I of the PrL subregion of mPFC (Fig. 5). This ratio in PrL subregion of mPFC in WKY (n = 3) and SHR animals (n = 3) were nearly equivalent 30.86 ± 6.07 (%) (mean ± SD) and 30.13 ± 9.23 (%), respectively. There was no significant difference in the percentage of TH-ir dopaminergic fibers between WKY and SHR (*p* > 0.05 from the Nested t-test).

## Discussion

The result of the current study reveals that in SHR brains (i) there is no dopaminergic cell loss in VTA, and (ii) there is no reduction of DA axons in the PFC. These results invalidate our hypothesis and provide evidence that dopaminergic innervation in PFC cannot account for ADHD-like behavior observed in these animals.

Despite the high prevalence of ADHD, the etiology of this neurodevelopmental disorder has not been fully elucidated, yet [63]. Evidence supporting the role of DA in the pathophysiology of ADHD comes from studies in wide-ranging areas [10, 12, 64, 65]. Nonetheless, studies on animal models aim at verifying the current postulate of a dysfunctional dopaminergic system in ADHD are scarce. The present results revealed the following: The mesocortical dopaminergic system appears to be normal in juvenile SHR, as suggested by (i) no alteration in the area density of TH-ir dopaminergic neurons in the VTA, (ii) no alterations in the volume density of TH-ir fibers in layer I of the PrL subregion of mPFC, (iii) no alteration in the percentage of TH-ir dopaminergic fibers in layer I of the PrL subregion of mPFC as revealed by subtraction of DBH-ir fibers from those that are TH-ir. To the best of our knowledge, this is the first study to evaluate the TH-ir neurons in VTA in the juvenile SHR to date. Similarly, the density of TH-positive fibers in the PRL in juvenile SHR and WKY was similar [66].

Although SHR is the most widely used model for ADHD, hypertension can be confounding [67]. To eliminate this confounding factor, we measured SBP in SHR and WKY. Juvenile SHR exhibited no hypertension, furthermore, there were no differences between the strains with respect to SBP, as previously described [68]. We also tested the face validity of the SHR and their usual control, WKY, in an infrared beam-based activity meter to examine global motor activity to validate that symptoms of ADHD appear before SHR develops hypertension. In behavioral comparison in the activity meter, SHR displays an increased total distance traveled when compared to WKY. Previous work showed that SHR traveled greater total distances in open field tests than did WKY rats [42, 56, 67, 69] which is interpreted as SHR rats present hyperactivity in motor function. These procedures have provided evidence that SHR mimics the basic behavioral characteristics of ADHD without having hypertension.

Since, TH is the rate-limiting enzyme of catecholamine biosynthesis, including DA, decreasing TH-ir density in mPFC may parallel reduced DA activity in the PFC, which produces hyperactivity in animals [70]. Contrary to expectation, we observed no significant changes in the density of TH-ir dopaminergic fibers in mPFC in SHR compared to WKY. This result is in line with one of the earlier finding that TH-ir fiber density did not differ in SHR in the PrL and Cg1 subregions of the mPFC in a 5-week-old SHR [66]. Our results are also in accordance with a study by Leo et al., which observed significantly lower TH mRNA levels in the mesencephalon in SHR only at P5 and at P7 [71] which can explain the lack of differences in our study at the postnatal fourth week. The lack of differences in TH-ir fiber density between groups in this study should not rule out the involvement of the mesocortical system in ADHD, since we have not assessed the DA neurotransmission. Future studies will be needed to assess the expression of DAT and DA receptors, DA vesicular storage, DA transporter density, and DA availability at postsynaptic receptors in SHR to better understand the molecular mechanisms that cause dopaminergic hypofunction in ADHD.

ADHD is subclassified according to symptom clusters, hyperactive/impulsive, inattentive, or combined [72] which may have a heterogeneous origin. Interestingly, the hyperactive/impulsive subtype of ADHD may be qualitatively different from the ADHD inattentive subtype [73]. It has been suggested that the inattentive subtype may result from dysfunction in the inhibitory action of the frontal cortex, whereas the hyperactive/impulsive subtype may arise from an impairment of subcortical structures such as substantia nigra and striatum [12, 73–76]. Substantia nigra plays an important role in movement and motor planning. The striatum serves as a mediator for many functions of the substantia nigra. The nigrostriatal dopaminergic pathway which projects from the substantia nigra to the striatum is closely linked with the striatum’s function [77, 78]. Hyperactivity in ADHD may result from excess dopaminergic activity in the striatum which is in line with increased striatal activity on PET in adolescents with ADHD relative to normal controls [79]. In our study, we found SHR displayed hyperactivity in the open field test, a result that is also similar to that of previous studies [56, 67, 69, 80, 81]. The cause of locomotor hyperactivity in the SHR might be related to altered dopaminergic functioning in the striatum, but this will remain speculation without further research.

In the present experiment, SHR was more active than WKY in the open-field test. Although hyperactivity is necessary; it is not sufficient for an animal model of a combined subtype of ADHD. Previous studies reported that SHR did not show any impairment of attention-related behavior in the 5-choice serial reaction time task [80] and a visual discrimination task [82]. We also showed no alteration in the TH-ir neurons in VTA and TH-ir fibers in mPFC in SHR. How should we interpret no alteration in the TH-ir neurons in VTA and TH-ir fibers in mPFC and the increased locomotor activity in the SHR? Perhaps, no alteration of the TH-ir fibers in the mPFC might be in line with previous findings that SHR does not display inattention symptoms of ADHD, and hyperactivity in the SHR might be a result of the dysregulation of dopamine in the striatum and SN. Even though the SHR is the most validated animal model of ADHD and shows some face validity, our data raises questions about the usefulness of the SHR as a model of the inattentive or combined subtype of ADHD. These results highlight that more research is required to further validate the use of SHRs as a suitable animal model for the inattentive or combined subtypes of ADHD as defined in DSM 5-TR. In this regard, previous findings should be treated with a degree of circumspection.

This study has limitations. First, as detailed characterization of the mesocortical dopaminergic fibers is not possible without classic anterograde or viral tracing, combining these techniques with multiple fluorescent immunohistochemistry would provide more precise anatomical information. Secondly, the use of different animals in immunohistochemistry and behavioral studies as those performed in different laboratories may prevent direct comparisons of the outcomes obtained from the two types of assessments. However, we used the same rat strains (SHR and WKY) from the same supplier (Charles River Laboratories) to obtain results that are directly comparable with each other. Thirdly, as substantia nigra and striatum are crucial parts of dopamine signaling and play a major role in motor control and attention, examining TH-ir neurons in the substantia nigra and TH-ir fibers in the striatum would provide in-depth information about the usefulness of the SHR as a suitable animal model for hyperactive/impulsive subtype of ADHD as defined in DSM-5-TR.

## Conclusions

Overall, with these limitations in mind, the present study demonstrated that SHR exhibits more hyperactivity than WKY rats. Also, SHR was normotensive at postnatal 4-weeks age. There were no anatomical changes in the mesocortical dopaminergic system of SHR in comparison with age-matched WKY suggesting complex interaction of dopaminergic neurotransmission is not limited to anatomical changes. In conclusion, we suggest that SHR might not be suitable animal model for the inattentive or combined subtypes of ADHD and future research is needed to disentangle the role of the mesocortical dopaminergic system in ADHD through other approaches.

## Methods

### Animals

Juvenile (4-week-old) male Spontaneously Hypertensive Rats (SHR) (n = 15) and Wistar Kyoto rats (WKY) (n = 13) (50–60 g on arrival, Charles River Laboratories, Wilmington MA) were used in the present study. All animals were acclimatized for 1 week before using them. Out of the total, 20 (SHR: n = 10; WKY: n = 10) and 8 rats (SHR: n = 5; WKY: n = 3) were used in behavioral and anatomical procedures, respectively. All animals were housed in a colony room with a 12-h light/dark cycle (light on at 8.00 am) and had ad libitum access to water and standard rat feed. All anatomical procedures were approved by the animal care and use committee of the University of Virginia and under the National Institutes of Health guidelines. The behavioral experiments were performed at Pamukkale University and under the approval of 2015/09. As common in SHR literature [15, 83, 84], only male rats were used in this study since ADHD-like symptoms are more prevalent in male rats [85], and ADHD is three times more prevalent in males than females [1, 86, 87]. Moreover, the same rat strain had behavioral testing in the present study which proved one of the main ADHD symptoms (increased locomotor activity) in SHR in the open field task. Furthermore, we measured the systolic blood pressure of animals prior to experiments to rule out the possible confounding factor i.e. hypertension.

### Blood pressure measurements

Animals had been trained to stay in the rat holder to condition the animal for this procedure which was repeated in 3 days and were tested at the beginning of the experiment. Noninvasive systolic blood pressure (SBP) measures obtained from day 4 were considered valid. Conscious rats were restrained in a warming chamber at 34 °C (RXRESTRAINER-S; BIOPAC BioPac Systems) for 10–20 min before non-invasive SBP measurements using a computerized indirect tail-cuff method (NIBP200A, BioPac Systems). The sensor (RXTCUFSENSOR9.5–9.5 mm; BioPac Systems), consisting of an infrared light source and infrared light detectors mounted in a 95 mm long inflatable rubber cuff, was placed on the base of the tail of the rats then SBP was recorded [88]. The mean SBP is an average of four measurements made over a 10-min period.

### Behavioral testing

All the animals, SHR (n = 10) and WKY(n = 10), were removed from the colony room and brought to the behavioral testing area in their home cages 30 min before the behavioral testing to minimize the stress due to exposure to a new environment. Behavioral testing was performed in a quiet room at the beginning of the light phase of the light/dark cycle. At the end of the test, the number of fecal boluses was counted, and the arena was cleaned with 70% ethanol prior to use to remove any scent clues left by the previous animal.

#### Locomotor activities in open field test

To validate the face validity of SHR, the animal was placed in the center, always facing the same direction, and allowed to explore the open field for 10 min. The locomotor activity was assessed using a computerized and automated activity monitoring apparatus (May Act 508, 42 × 42 × 42 cm; Commat Ltd, Ankara, Turkey) capable of tracking different behavioral activities. The apparatus comprised a black floor divided into 196 equal squares and surrounded by a transparent wall equipped with infrared sensors. Multiple variables were measured (distance moved, immobility, horizontal, vertical, and stereotypy movements).

### Tissue preparation and sampling design

The processing of tissue from each of these eight rats used for anatomical studies was as follows: sections from 8 brains (5 SHR, 3 WKY) were used for single immunolabeling for TH, light microscopy, and sections from 6 brains (3 SHR and 3 WKY) were used for dual immunolabeling for dopamine DBH and TH, confocal microscopy. The TH-ir was considered to be the most optimal marker to identify dopaminergic neurons [89] and fibers [90, 91]. However, because cortical noradrenergic fibers also express TH [92], DBH immunoreactivity is widely utilized as a specific biomarker for cortical noradrenergic fibers[93]. We employed double-labeling techniques with anti-TH and anti-DBH antibodies to determine whether the TH-ir fibers do indeed selectively label dopaminergic fibers in mPFC.

The rats were deeply anesthetized with an overdose of euthosole (excess of 150 mg/kg, i.p) and transcardially perfused using room-temperature Tyrode’s solution (137 mM NaCl, 5.5 mM Dextrose/Glucose, 1.2 mM MgCl_2_, 2 mM KCl, 0.4 mM NaH_2_PO_4_, 0.9 mM CaCl_2_, 11.9 mM NaHCO_3_, in 1 L filtered dH_2_0) followed by 4% paraformaldehyde in 0.1 M phosphate buffer (PB; pH 7.4). Brains were removed and allowed to postfix overnight in 4% paraformaldehyde at 4 °C. Four sets of coronal vibratome sections through the mPFC and VTA cut at 50 μm were collected in PB. All morphological analyses were done in sections obtained through the rostrocaudal extent, between Bregma 4.20 and 2.76 for mPFC and Bregma -5.52 and -6.00 for VTA, according to Stereotaxic Rat Atlas [94]. Adjacent sections were mounted on glass slides and stained with Nissl to determine the borders of the region of interest. Sections that were not processed immediately were treated with 1% sodium borohydride (NaBH_4_; 1 g NaBH_4_ in 100 ml 0.1 M PB) to stop fixation, rinsed until the bubbles had cleared and stored in 0.05% sodium azide in phosphate-buffered saline (PBS) at 4 °C.

### Immunocytochemistry

Tissue sections were collected in PBS and blocked in 1% bovine serum albumin (BSA; Sigma Aldrich) in 0.01 M PBS, pH 7.4, for 30 min at room temperature. Free-floating tissue sections were then transferred in a chicken polyclonal antibody against TH (Abcam, Cat# AB76442, 1:1000; Table 2) and 1% BSA in 0.01 M PBS with 0.05% NaN_3_ and 0.3% Triton X-100 (Sigma Aldrich) for incubation of 1–3 days. The sections were then rinsed in 0.01 M PBS and transferred into biotinylated goat anti-chicken secondary antibody (Vector Laboratories, Burlingame, CA; Cat# BA-9010; 1:100 dilution) for 2 h, followed by ABC-DAB visualization. All sections were mounted serially on gelatin-subbed slides, dehydrated, and coverslipped using DPX mounting media (Sigma Aldrich, St. Louis, MO).

**Table 2:** List of antibodies and dilutions used in the study

Name	Host	Antigen characteristics	Source	Dilution
Anti-Tyrosine hydroxylase	Chicken (polyclonal)		Abcam, AB76442;	1:1000
Anti-Dopamine Beta hydroxylase	Mouse (monoclonal)	Clone 4F10.2 (Milstein 2007)	Millipore Co. Temecula, CA, MAB308	1:300
Anti-Chicken secondary antibody	Goat	Chicken IgG, conjugated to biotin	Vector Laboratories, Burlingame, CA; BA-9010	1:100
Anti-Mouse secondary antibody	Donkey	Mouse IgG, conjugated to rhodamine (TRITC)	Jackson Immunoresearch Laboratories, Inc., West Grove, PA; #715-025-151	1:500
Anti-Chicken secondary antibody	Donkey (polyclonal)	Chicken IgG, conjugated to Alexa Fluor 488	Jackson Immunoresearch Laboratories, Inc., West Grove, PA, #: 703-545-155	1:500

The table summarizes all antibodies used and their species specificity, dilutions, and sources

Sections for dual-labeling with two markers were incubated in a blocking solution containing 1% bovine serum albumin in PBS for 30 min. The sections were then transferred into an antibody cocktail that contained 1:300 monoclonal anti-DBH (Millipore Co. Temecula, CA, catalog no MAB308; 1:300) and 1:1000 chicken polyclonal anti-TH (Abcam, Cat# AB76442, 1:1000), 0.05% NaN_3,_ 0.3% Triton X-100, 1% BSA in PBS, and they were incubated at room temperature for 48 h. Then the sections were rinsed three times in PBS (5 min each), and incubated in a mixture of donkey anti-mouse secondary antibody conjugated with rhodamine (TRITC) (red, Jackson Immunoresearch Laboratories, Inc., West Grove, PA; catalog no: 715-025-151,1:500) and polyclonal donkey anti-chicken secondary antibody conjugated with Alexa Fluor 488 (green, Jackson Immunoresearch Laboratories, Inc., West Grove, PA, catalog no: 703–545-155, 1:500) in PBS for 2 h at room temperature in the dark. They were then rinsed three times in PBS and mounted on gelatin-coated glass slides, air-dried, and covered with an anti-fading mounting medium (Vector Laboratories, Burlingame, CA; catalog no. H-1000). The brain sections from two experimental groups were processed at the same time using the same preparations of the incubation solutions to eliminate variability due to protocol handling.

### Image acquisition

The sections processed for light microscopy were examined on a Leica DMLB microscope equipped with a digital camera (Leica MC170 HD). Low magnification images were taken with the 4x, and 10 × objective lens, and high magnification images were taken with a 40 × objective lens for quantitative analysis of TH-ir structures. The images were then adjusted for contrast and exposure in Adobe Photoshop. Architectural borders of VTA and layer I of the prelimbic (PrL) subregion of the mPFC (ROI) were identified after matching tissue sections with comparable atlas sections. These borders were also superimposed on adjacent sections that were stained for TH**.** Histoarchitectural transitions in the VTA and PrL subregion of the mPFC were examined in TH-stained sections. We obtained counts from both hemispheres, and all data for every analysis were pooled.

#### Area density of TH-ir neurons in VTA

Quantitative data were collected by one experimenter blinded to the animal experimental status using an unbiased sampling approach: We measured the volume density of TH-ir dopaminergic neurons in the VTA, using the Stereo Investigator (MBF Bioscience, Inc.) software. For this, the coronal sections that contained VTA were identified for analysis; these sections were comparable to plates # 79–82 of the rat brain atlas [94]. One-fourth of the sections from each brain were selected in accordance with systematic random sampling protocols, and four sections were used for counting cells [95]. Using the ‘outline’ tool of the Stereo Investigator, the contour of the VTA was drawn under low magnification (4 × objective). The selected areas were similar for all animals. Then, the outlined region was overlaid with a random series of counting frames [96, 97]. The TH-ir cell nuclei counts were performed under high magnification (40X). The top and bottom 5 µm at the top and bottom of the 50 μm thick sections were considered exclusion zones to reduce prevent over or underestimation related to surface artifacts. When the nucleus of a TH-ir neuron was unambiguous within the counting frame (50 × 50 μm), it was included in the cell count. In a range of 53 and 89 counting frames in four sections were evaluated per animal. Each counting frame consisted of two exclusion lines (left and bottom edges) and two inclusion lines (right and top edges). TH-ir cell nuclei were counted if they were found entirely within a counting frame or transected by at least one of the inclusion lines of a counting frame but not any of the exclusion lines of the same counting frame [98]. The area TH-ir dopaminergic neuron density (N_A_) in the ROI was calculated using the following formula: N_V_ = N/A_ROI_ where N is the total number of TH-ir neurons and A_ROI_ is the total area analyzed (number of counting frame x area of counting frame) [97, 99].

#### Fiber density analysis using brightfield microscope

In order to quantify the density of TH-ir fibers, we outlined the PrL subregion of the mPFC using landmarks derived from Paxinos and Watson [94]. The ROI was outlined under low magnification (4×) and a 150 μm wide measurement frame is positioned perpendicular to the cortical surface, at 10×magnification. Six different measurement frames per animal were used. Using a 40× objective, each TH-ir fiber in the ROI was traced, and the total length of fibers was computed using Neurolucida® software (MBF Bioscience, Inc.). The area of the measurement frame was also computed. The fiber density (μm/μm^3^) in each measurement frame was determined by dividing the total TH-ir fiber length over the measurement frame box volume. The volume of the measurement frame box on each image was obtained by multiplying the area of the measurement frame box with the thickness of the section.

#### Fiber density analysis using confocal microscopy

Fibers were imaged using an 80i microscope fitted with a C2 scanning system (Nikon Instruments, Inc., Melville, NY) and a 10 × objective (Nikon, CFIPlanApo; NA = 0.45). The fibers were matched for the wavelengths of the two lasers in the system (argon laser, 488 nm, 10 mW, Alexa Flour 488; DPSS laser, 561 nm, 10 mW, TRITC). A 60 × objective (Nikon, PlanApo VC; NA = 1.4) was used with a multi-track scanning method to completely separate the detection of the Alexa 488 and TRITC signals. A total of 3 SHR and 3 WKY animals were used and 9 images per animal were quantified. More precisely, 3 consecutive sections were imaged for the PrL subregion of the mPFC, and 3 images were acquired per section. All images were taken in lamina I. To avoid experimental bias, the experimenter was blinded to the experimental groups. For each image acquisition, the pixel size (0.21 μm), dwell time (10.8 μm), step size (0.25 μm), and z slice (5 μm) were the same. Each confocal image corresponded to a field of 1024 × 1024 pixels Photomicrographs for figures were made from maximum intensity images of the confocal stacks of single physical sections and were exported as a TIFF file to be analyzed with an image analysis system (Image-Pro Plus 7, Media Cybernetics, Inc, Silver Spring, MD). Only adjustments of brightness and contrast were used for images used in photomicrographs. The total length of TH-ir and DBH-ir fibers were evaluated in ROI with Image-Pro Plus 7 (Media Cybernetics, Inc, Silver Spring, MD) using manual tracing. The percentage of TH-ir fibers that were not also DBH-ir was determined by dividing the total TH-ir fiber length over the total fiber length.

### Statistical analysis

Data analysis was performed using Microsoft Excel 2007and and GraphPad Prism (GraphPad Software, San Diego, CA, USA) software. All graphs and figures were created using Prism (9.3.1; GraphPad Software, San Diego, CA, USA) and Adobe Photoshop CS5 (23.2.1), respectively. Descriptive data were obtained by using descriptive statistical methods such as mean, standard deviation, and median and submitted to the Shapiro–Wilk normality test. All data displayed a normal distribution. For analysis of SBP data and open field data (distance moved, immobility, horizontal, vertical, and stereotypy movements) was conducted using an unpaired t-test. For immunohistochemical analysis, to compare the density of TH-ir neurons and fibers between groups, we used the Nested-t test. All statistical tests are two-tailed at a significance level of 0.05. Data on graphs represent mean ± standard deviation (SD). For all figures, ***p < 0.001, **p < 0.015, *p < 0.05.

## Data Availability

The raw data supporting the conclusions of this manuscript will be made available by the authors, without undue reservation, to any qualified researcher.

## Abbreviations

ADHD: Attention deficit hyperactivity disorder
DA: Dopamine
DBH: Dopamine β-hydroxylase
mPFC: Medial prefrontal cortex
NE: Norepinephrine
PFC: Prefrontal cortex
PrL: Prelimbic area
SHR: Spontaneously hypertensive rat
TH: Tyrosine hydroxylase
VTA: Ventral tegmental area
WKY: Wistar kyoto rat
